# Alzheimer's Association and ASNR announce recipients of imaging grants

**DOI:** 10.1002/alz.13743

**Published:** 2024-02-21

The Alzheimer's Association and The Foundation of the American Society of Neuroradiology (ASNR) have announced the recipients of a new funding program for imaging‐related research. The goal of The Alzheimer's Association & The Foundation of the American Society of Neuroradiology Funding Opportunity: Imaging Research in Alzheimer's and Other Neurodegenerative Diseases is to support the development of new novel imaging tools, advance imaging‐based research, and provide funding for novel and innovative imaging data analysis pertaining to Alzheimer's and related dementia.

Awardees, who each receive up to $200,000 for the 1‐year period of funding are:

Min‐Jeong Kim, MD, PhD, assistant professor of psychiatry and behavioral health at the State University of New York at Stony Brook and a faculty member at its Neurosciences Institute, for the study, “PET Imaging of Neuroinflammation in Dementia with Lewy Bodies.”

Anouk den Braber, PhD, assistant professor of behavioral and movement sciences, biological psychology, and personalized medicine at the Amsterdam University Medical Center (VUMC), for the study, “Genetic and Environmental Contributions to Longitudinal Tau Trajectories.”

Phillip Zhe Sun, PhD, professor of radiology and imaging sciences at Emory University in Atlanta, for the study, “Development of CEST MRI for Dementia with Lewy Bodies.”

For more information about this and other funding opportunities, visit alz.org/grants.

## Attend an ISTAART “Basics of” webinar this March and April

The Alzheimer's Association® International Society to Advance Alzheimer's Research and Treatment (ISTAART) Professional Interest Areas (PIAs) will be hosting “Basics of…” webinars throughout March and on April 1. Each webinar will provide educational content from experts in Alzheimer's and dementia research that can stand alone or be paired with AAIC educational sessions in a flip‐classroom format to help early career researchers form foundational knowledge on key concepts. These sessions will include a 20‐minute primer presentation, quiz, and Q&A. The webinar schedule is below. For additional information, email ISTAART@alz.org.

**Friday, March 1**

9:00–10:00 am CT

Neuroimaging PIA

The Basics of Neuroimaging: Translational Artificial Intelligence

Speaker: David Jones

Moderator: Michael Ewers

Panelists: Daniel Alexander, Christos Davatzikos, Alexandra Young

Register here: https://alz‐org.zoom.us/webinar/register/WN_kIOc4d9cTJmByONPmm‐IFQ

**Monday, March 4**

10:30–11:30 am CT

Technology and Dementia PIA

The Basics of Artificial Intelligence in the Context of Dementia Care

Speaker: Sudeshna Das

Moderator: Ian Cleland

Panelists: Colby Ford, Mariana Pinheiro Bento

Register here: https://alz‐org.zoom.us/webinar/register/WN_bzlgERbSRNyIfZ5eMveVdg

**Tuesday, March 5**

11:00 am–12:00 pm CT

Atypical Alzheimer's Disease PIA

The Basics of MRI for Atypical Alzheimer's Diagnosis

Speaker: Juhan Reimand

Moderator: Colin Groot

Panelists: Rosaleena Mohanty, Jennifer Whitwell

Register here: https://alz‐org.zoom.us/webinar/register/WN_InJNIY‐6SUmEcKXSsCeXdA

**Monday, March 11**

1:00–2:00 pm CT

Perioperative Cognition and Delirium PIA

The Basics of Perioperative Neurocognitive Disorders

Speaker: Lisbeth Evered

Moderator: Niccolo Terrando

Panelists: Emma Cunningham, Esther Oh

Register here: https://alz‐org.zoom.us/webinar/register/WN_ajqTyePPTM69NpH‐Uw_8Xg

**Wednesday, March 13**

10:00–11:00 am CT

Biofluid‐Based Biomarkers PIA

The Basics of Fluid Biomarker Development for Clinical Implementation

Speaker: Mircea Balasa, Olivia Belbin

Moderator: Yanaika Hok‐a‐Hin

Panelists: Jeff Dage, Marta del Campo

Register here: https://alz-org.zoom.us/webinar/register/WN_A3A3a0yCT3uxV4q6QoVHAA

**Thursday, March 14**

10:00–11:00 am CT

Sensory Health and Cognition PIA

The Basics of Why Senses Matter for Cognitive Health and Dementia Risk

Speaker: Natalie Phillips

Moderator: Carrie Nieman

Panelists: Nathalie Giroud, Natascha Merten

Register here: https://alz‐org.zoom.us/webinar/register/WN_U‐gSjXbwRzCg_ZgiUYpIXQ

**Friday, March 15**

10:00–11:00 am CT

Sensory Health and Cognition PIA

The Basics of Considering the Senses in Your Research

Speaker: Heather Whitson

Moderator: Walter Wittich

Panelists: Ingrid Ekström, Carrie Nieman, Esther Oh, Natalie Phillips

Register here: https://alz‐org.zoom.us/webinar/register/WN_rHbvcLPgSa2H_SEycvWNDg

**Tuesday, March 19**

9:30–10:30 am CT

Electrophysiology PIA

The Basics of Electrophysiology: EEG, MEG & Neurophysiology

Speaker: Aina Puce

Moderator: Bahar Güntekin

Panelists: Mihály Hajós, Yang Jiang

Register here: https://alz‐org.zoom.us/webinar/register/WN_6nj‐i9kDR2OJqRI9VPOQPw

**Wednesday, March 20**

10:30–11:30 am CT

Neuromodulatory Subcortical Systems PIA

The Basics of Neuromodulatory Subcortical Systems: Cholinergic System

Speaker: Taylor Schmitz

Moderator: Heidi Jacobs

Panelists: Michel Grothe, Tessa Harrison

**Thursday, March 21**

2:00–3:00 pm CT

The Basics of Neuroimaging Data Harmonization (Hosted by the Alzheimer's Disease Sequencing Project)

Speaker: Shannon Risacher

Moderator: Timothy Hohman

Panelists: Randa Melhem, Shannon Risacher, Sindhuja Tirumalai Govindarajan

Register here: https://alz‐org.zoom.us/webinar/register/WN_Cn_UlRHqTP2zXSzmFf0‐5Q

**Friday, March 22**

1:30–2:30 pm CT

The Basics of Participant Recruitment: Building for Inclusion

Speaker: Kathryn (Kady) Nearing, Whitney Wharton

Moderator: Erika Tarver

Panelists: Cerise Elliott, Kathryn (Kady) Nearing, Whitney Wharton

Register here: https://alz‐org.zoom.us/webinar/register/WN_cSky57v6RaG‐nvlNs3SdIw

**Wednesday, March 27**

10:00–11:00 am CT

The Basics of Multimorbidity and Dementia

Speaker: Lucy Stirland

Moderator: Ana Quiñones

Panelists: Thiago Avelino‐Silva, Shirine Moukaled, Preeti Zanwar

Register here: https://alz‐org.zoom.us/webinar/register/WN_iDCrnzd7SamWjq6O7Dqt0A

**Thursday, March 28**

10:00–11:00 am CT

Reserve, Resilience, and Protective Factors PIA

The Basics of Reserve and Resilience: Concepts, Methodologies and Scientific Evidence

Speaker: Eider Arenaza‐Urquijo

Moderator: Elisa Resende

Panelists: Brian Gold, Lauren Grebe

Register here: https://alz‐org.zoom.us/webinar/register/WN_3g25pu8jQQ2WdAUy5cE95g

**Monday, April 1**

10:00–11:00 am CT

Sleep and Circadian Rhythms PIA

The Basics of Circadian Rhythm Changes in Aging and Dementia

Speaker: Derk‐Jan Dijk

Moderator: Laura Stankeviciute

Panelists: Paula Desplats

Register here: https://alz‐org.zoom.us/webinar/register/WN_X14hpbllTR‐8PutZqXin5A

Visit alz.org/ISTAARTbasics to see more events in this series.

